# Schedule-based Family-centered Rounds: A Novel Approach to Achieve High Nursing Attendance and Participation

**DOI:** 10.1097/pq9.0000000000000265

**Published:** 2020-03-13

**Authors:** Alaina K. Kipps, Marisa S. Albert, Sean Bomher, Shirley Cheung, Shannon Feehan, Joseph Kim

**Affiliations:** From the * Department of Pediatrics, Stanford School of Medicine, Palo Alto, Calif.; † Department of Medical Affairs, Lucile Packard Children’s Hospital, Palo Alto, Calif.; ‡ Department of Process Improvement, Lucile Packard Children’s Hospital, Palo Alto, Calif.; § Department of Nursing, Lucile Packard Children’s Hospital, Palo Alto, Calif.

## Abstract

**Introduction:**

Bedside nurse (RN) presence during family-centered rounds (FCR) enhances communication and collaboration for safer, higher-quality care.1–3 At our institution, RN participation in FCR was variable and lower than desired. The content discussed at each bedside during rounds was inconsistent, contributing to the irregular achievement of established FCR checklist items.

**Methods:**

Using a scheduling tool with a prioritization algorithm and set time allotment/patient, we implemented schedule-based family-centered rounds (SBFCR) on a pediatric acute care unit. Primary outcome metrics included RN attendance and participation. We tracked rounding checklist compliance, parent presence on rounds, and adherence to the schedule. Surveys provided information on provider and family satisfaction. Perceived impact on teaching was the balancing measure because the structure discouraged spending extra time at the cost of team tardiness for the next patient.

**Results:**

We created a schedule for 95% workweek days, with the rounding order kept for 93%. Mean RN attendance increased from 69% to 87% and participation increased from 48% to 80% with SBFCR (*P* < 0.001 for each). FCR checklist compliance increased from 60% to 94% (*P* < 0.001). Families felt more informed and able to attend; their presence at rounds rose from 66% to 85% (*P* < 0.001). Most faculty and trainees felt SBFCR was efficient and observed increased teaching with SBFCR.

**Conclusions:**

SBFCR provides an organizational framework for increased RN attendance and participation as well as greater family presence during rounds. The system elevated provider satisfaction with rounding without degrading the perceived educational experience.

## Abstract 2

Supplemental Digital Content is available in the text.

## INTRODUCTION

Bedside family-centered rounds (FCR) have been standard practice for medical teams at Lucile Packard Children’s Hospital Stanford for several years. FCR, central to family-centered care, aims to encourage shared decision making between families and providers.^4,5^ Medical rounds must accomplish several functions, including creating and clarifying the plan of care for the family and interdisciplinary team, discharge preparation, and trainee teaching. Our multidisciplinary teams aim for “one-message-one-time” during morning rounds.^6^

Bedside nurses (RNs) are key members of the healthcare team and carry out the daily plan while serving as the central point of communication for the patient and family. RNs are uniquely positioned to provide real-time patient information and detect and report out clinical changes due to their frequent contact with the patient.^7^ The presence of nursing during FCR is critical for effective communication that contributes to high quality and safer patient care.^1–3,8^ Rounds, however, are just one of many morning tasks for RNs caring for multiple patients.

Despite a culture of valuing RN presence during rounds, local audits of RN involvement in FCR revealed inconsistent and variable attendance. Additionally, the content presented for each patient was inconsistent, resulting in the variable accomplishment of rounding tasks. The variability in nursing attendance, waiting for RNs and interpreters, and lack of standard rounding content prolonged rounds and led to high levels of provider frustration. Additionally, some staff raised concerns that rounding without the RN would lead to care delays and potential safety issues.

Our primary aims were to increase RN attendance from <50% to >85% and participation from <40% to >75% in daily rounds within 6 weeks of implementation. We hypothesized that creating and adhering to a structured schedule for rounding would enable RNs to anticipate and thereby participate in FCR.^9^ Secondary aims included the increased family presence during rounds, complying with standardized presentation format including checklist item discussion, and increasing medical team satisfaction with the rounding process.

## METHODS

We conducted this project in 2 phases. Phase I began with the implementation of the primary intervention, schedule-based family-centered rounds (SBFCRs). Phase II began when family members were informed daily of the scheduled window for rounding on their child. This study was reviewed and determined to be a quality improvement project by the Stanford University Institutional Review Board.

### Context

We initiated this project on a 20-bed pediatric acute care cardiology unit of a 311-bed freestanding academic quaternary care children’s hospital. Nursing ratios are 3:1 for telemetry patients and 4:1 for all other patients. The cardiology service began traditional FCR in 2012. Historically, RNs were encouraged to participate in FCR.

In addition to the RN, the rounding team includes the pediatric advanced cardiac therapies (PACTs) and general cardiology (GC) attendings, the pediatric cardiology fellow, a pediatric resident, advanced practice providers, a pharmacist, dietician, and case manager. Consultants and ancillary team members such as interpreters or social workers were paged once the team was at the bedside. Rounding order, apart from grouping patients by service (PACT or GC), was typically by room number, and calls to each patient’s RN were made only minutes before commencing rounds. Our RNs are part of a union that specifies break requirements. Morning breaks often coincide with rounds. We made the schedule accessible to all staff for RNs and ancillary team members to structure their morning activities in anticipation of when they needed to be present for rounds.

### Planning the Intervention

**Fig. 1.**
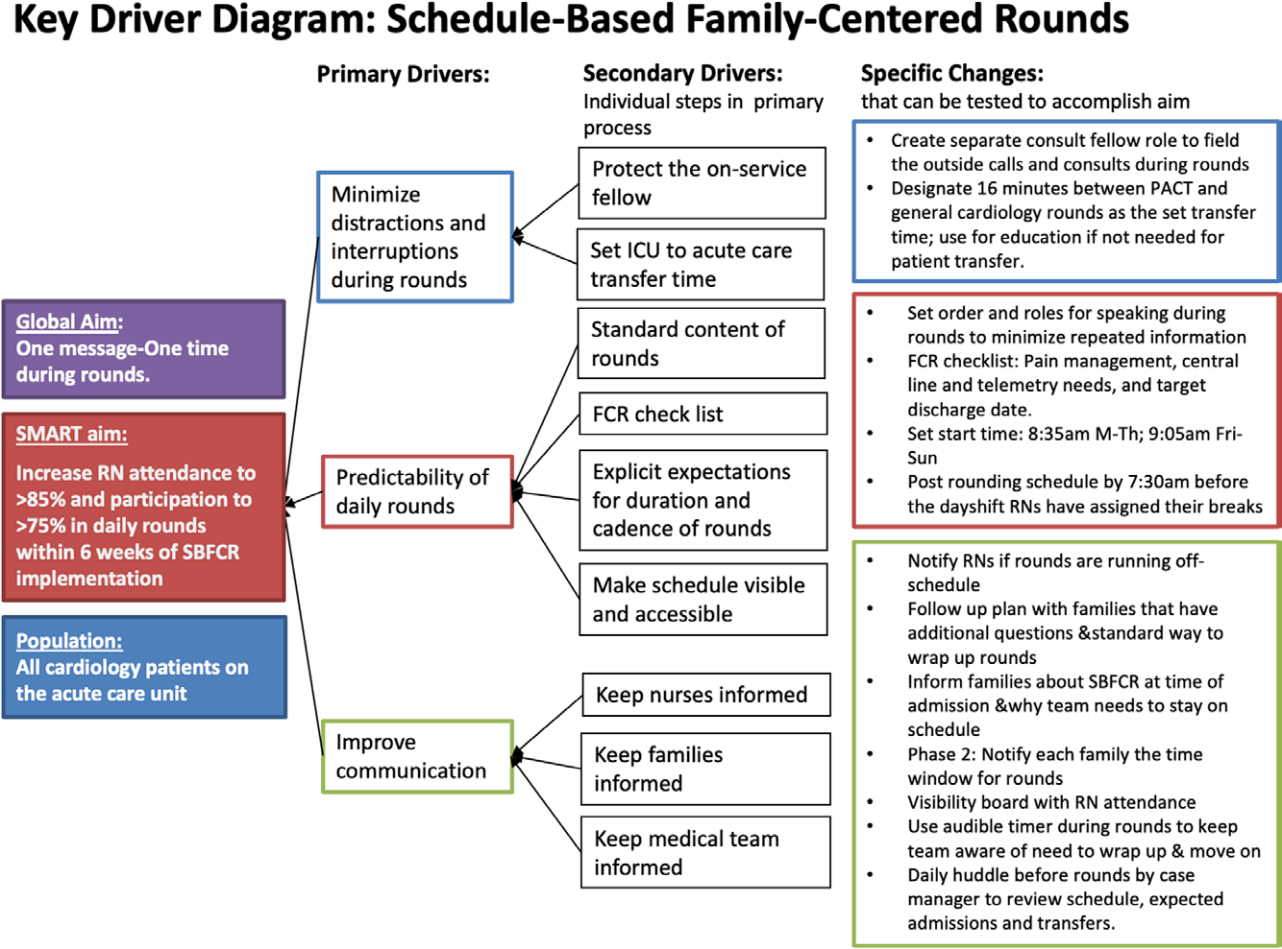
Project key driver diagram. FCRs, family-centered rounds; ICU, intensive care unit; RNs, nurses; PACTs, pediatric advanced cardiac therapies; SBFCRs, schedule based family-centered rounds.

The improvement team included a process improvement expert, the unit nursing management team and medical director, the case manager, a family advocacy representative, and administrative leaders. Stakeholder interviews of nurses, advanced practice providers, trainees, attendings, and ancillary members were held to understand each group’s needs for a predictable FCR process. We developed a key driver framework during interviews, observation of rounds, and design sessions (Fig. 1) and aligned on a vision for our primary intervention, SBFCR.^10^

To establish a reliable daily schedule for rounds, the improvement team set a standard start time and per-patient time allotment. Using a time-motion methodology described by Bhansali et al.,^11^ times were established in early 2015 after observation of 128 discrete rounding encounters over a convenience sample of 8 weekdays. The improvement team developed a prioritization scheme to determine how patients were scheduled. Priority was given to acutely ill patients, then grouped by service, time of discharge, and need for interpreter services. The SBFCR checklist helped teams cover all key elements in the time allotted on the schedule (see Family Centered Rounds for Each Patient: In order, by roles with associated content, and FCR checklist. See **Supplemental Digital Content 1**, http://links.lww.com/PQ9/A163).

### The Intervention

Each morning by 7:30 am, the case manager and fellow created and posted the schedule for nurses to plan morning breaks and patient care activities around rounds for their specific patients. All team members, including ancillary staff and consultants, had access to the schedule saved to a secure cloud storage site. PACT encounters defaulted to 10 minutes/patient and GC encounters to 8 minutes/patient with no extra time scheduled between patients. The medical team then had the option to flex up or down the duration of time allotted per patient before posting the final schedule. On rounds, an audible cue signaled when 2 minutes remained in a patient’s allotted time. If the team finished an encounter early, they used the extra time between patients for order entry or teaching. We implemented a standard plan to get back on schedule in the case of delays: if the team was behind >4 minutes, the team skipped the next patient and resumed SBFCR with the following patient. The skipped patient was rounded on later during built-in buffer time (16 min between PACT and GC patients) or at the end of scheduled patients.

### Implementation Timeline

We collected preintervention data on RN attendance from May 11, 2015, to July 2, 2015. Preimplementation surveys of providers were conducted in May 2015. Phase I began July 13, 2015, with the implementation of all the SBFCR components during the workweek. The 14-month intervention was continued to September 30, 2016. We communicated adherence to SBFCR with a visibility board updated by case management and nursing leaders. Nurses who missed rounding encounters were interviewed to understand barriers to full participation. Weekly feedback sessions with the improvement team and rounding team were held to facilitate subsequent Plan-Do-Study-Act (PDSA) cycles. By September 2015, on request from RNs, SBFCR expanded to include weekends (PDSA 1). Consistently, RNs assigned to patients with ventricular-assist devices accompanied them to hospital school at 9:00 am, thus missing later rounds. In December 2015, the case manager adjusted the schedule before finalizing it to move these patients before 9:00 am (PDSA 2), and family prephase II surveys were collected. Phase II began January 2016 with patient/family notification of rounding window (± 15 min of the specific scheduled time) and educating families about SBFCR (Family education brochure describing SBFCR. See **Supplemental Digital Content 2**, http://links.lww.com/PQ9/A164). In February 2016, we implemented a daily huddle before rounds to review the schedule and anticipated admissions (PDSA 3). We conducted postimplementation surveys for providers during March 2016, and for families in June 2016. After 14 months, following completion of data collection, the scheduling algorithm was adapted into an electronic health record tool to help automate the schedule creation (for an example of SBFCR schedule as created in EPIC web tool, see **Supplemental Digital Content 3**, http://links.lww.com/PQ9/A165). Once published, anyone can access the schedule from within the electronic health record. This intervention has facilitated sustainability by streamlining the scheduling process, enabling the spread of SBFCR to other acute care services.

### Measures

**Fig. 2.**
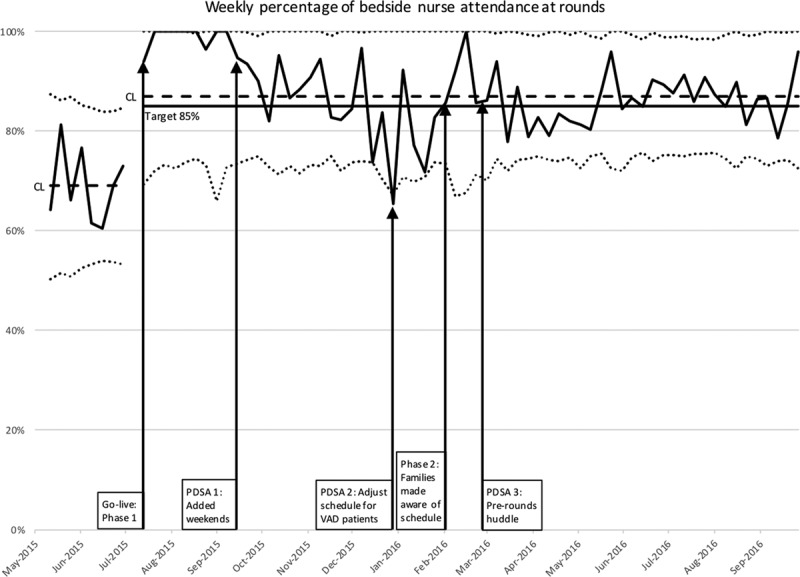
Weekly percentage of bedside nurse attendance at rounds. *P*-chart showing May 2015 through September 2016 with percent weekly nurse attendance. Control limits shown at 5% and 95% CI in dotted lines with the centerlines (CLs) marked in dashed lines and target in solid. Phase I and II and 3 Plan-Do-Study-Act (PDSA) cycles are noted on the timeline. CI, confidence interval.

**Fig. 3.**
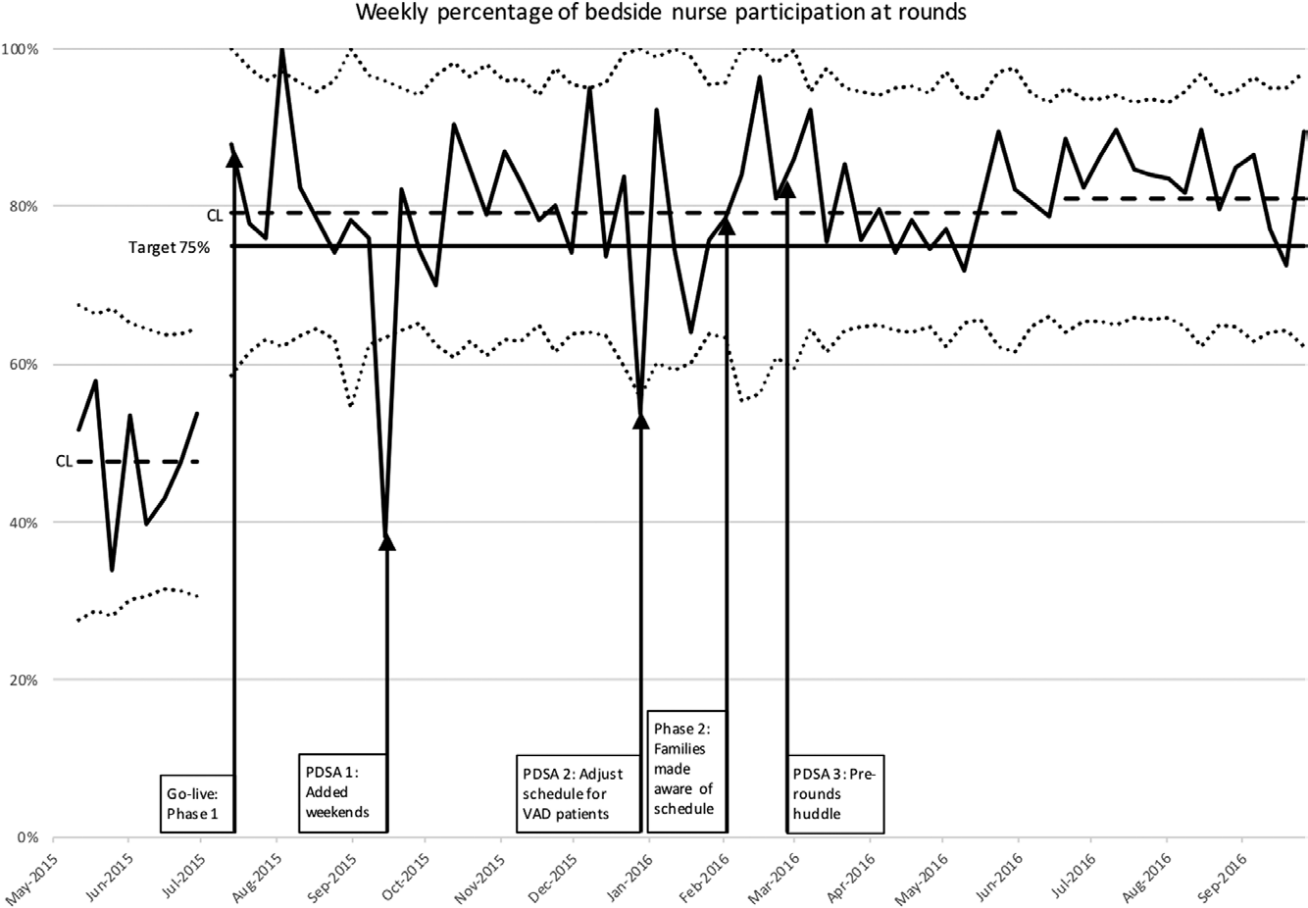
Weekly percentage of bedside nurse participation at rounds. *P*-chart showing May 2015 through September 2016 with percent weekly nurse participation. Control limits shown at 5% and 95% CI in dotted lines with the centerlines (CLs) marked in dashed lines and target in solid. Phase I and II and 3 Plan-Do-Study-Act (PDSA) cycles are noted on the timeline. CI, confidence interval.

The primary outcome measures were RN attendance and RN participation. We defined attendance as being present to listen to rounds and participation as the bedside RN delivering the patient introduction, overnight events and vital signs, and his/her concerns. The case manager collected data from Monday to Friday, which included FCR start and end time, number of patients, and whether the team adhered to the scheduling order. RN attendance and participation, family member presence, FCR checklist items (pain management discussion, central line and telemetry monitoring needs, and target discharge date), and deviations between expected and actual rounding time were tracked for every patient. *P*-charts were made to track our primary aim with average weekly % nurse attendance (Fig. 2) and % nurse participation (Fig. 3).^12^ We examined in detail one random week per month to assess the team’s compliance with keeping SBFCR patient order and timeliness and how these features affected RN and family participation. Additionally, nursing management tracked RN attendance daily on the unit’s visibility board with notes on obstacles to rounds participation. These barriers were reviewed weekly by the multidisciplinary improvement team to inform new interventions to trial.

We conducted surveys to measure provider and RN satisfaction with the rounding process before and 8 months after implementation. These surveys were modified from surveys used during routine FCR implementation at our hospital. Due to small group sizes and desire for anonymity, we combined responses from trainees and nurse practitioners. The RN surveys focused on the impact of rounds attendance on care coordination and communication. We assessed attending physician, trainee, and nurse practitioner perception of the effect on teaching on the postimplementation survey as a balancing metric; the majority had experienced cardiology rounds before and after SBFCR. Families were surveyed before and after phase II. All survey questions used a Likert scale for responses as follows: (1) strongly agree; (2) agree; (3) neutral; (4) disagree; and (5) strongly disagree. We considered a positive response as “agree” or “strongly agree.”

## RESULTS

During the study, we conducted 5,495 rounding encounters. We found stability in surgical program volumes, the heart failure-transplant program, and hospital case mix index across preintervention and postintervention periods.

### Nursing Presence and Participation in Rounds

During the preintervention period, data on 578 rounding encounters were captured. Data collected for 92% of weekdays during the 14-month intervention period recorded 3,532 rounding encounters (the remaining 1,963 encounters fell on weekends, holidays, or when the case manager was absent). Mean RN attendance increased from 69% to 87% after SBFCR implementation (Fig. 2, *P* < 0.001). Mean RN participation increased initially from 48% to 79% for 11 months and then rose to 81% from June 2016 onward (Fig. 3, *P* < 0.001). Adjustments made to SBFCR (PDSA 1–3 and phase II) did not change mean RN attendance and participation significantly. The mean time per patient at the bedside rose from 7.6 to 8.6 minutes, *P* < 0.001.

### Intervention Compliance

A quarter of these 3,532 rounding encounters were evaluated to assess team compliance with the intervention and effect on our primary outcome metrics. Audits of 861 specific rounding-encounters over 63 days collected during a random week/mo revealed the team held rounds in the scheduled order for 93% of patients (n = 804). In 696 encounters (87%), the RN attended rounds; in 634 (79%), the RN participated in rounds. When the team rounded out of scheduled order (n = 57), the RN attendance fell to 77%, and participation decreased to 68% (*P* value = 0.037 and 0.047, respectively, compared to % when in order). The medical team began rounds within 5 minutes of the scheduled time in 53% of these 861 encounters. Timeliness within 5 minutes of the exact scheduled time did not affect RN attendance or participation. For phase II, we assessed timeliness to the rounding window given to parents. The team rounded during this window for 83% of encounters.

### Standardization

A secondary goal was to increase compliance with the standardized FCR presentation format and checklist review. Rounding checklist compliance increased from 60% preimplementation to 90% within one month of phase I. Over the next year, September 2015 to September 2016, compliance rose and sustained at 94%; *P* < 0.001 compared to prephase I.

### Provider and Nurse Satisfaction

**Fig. 4.**
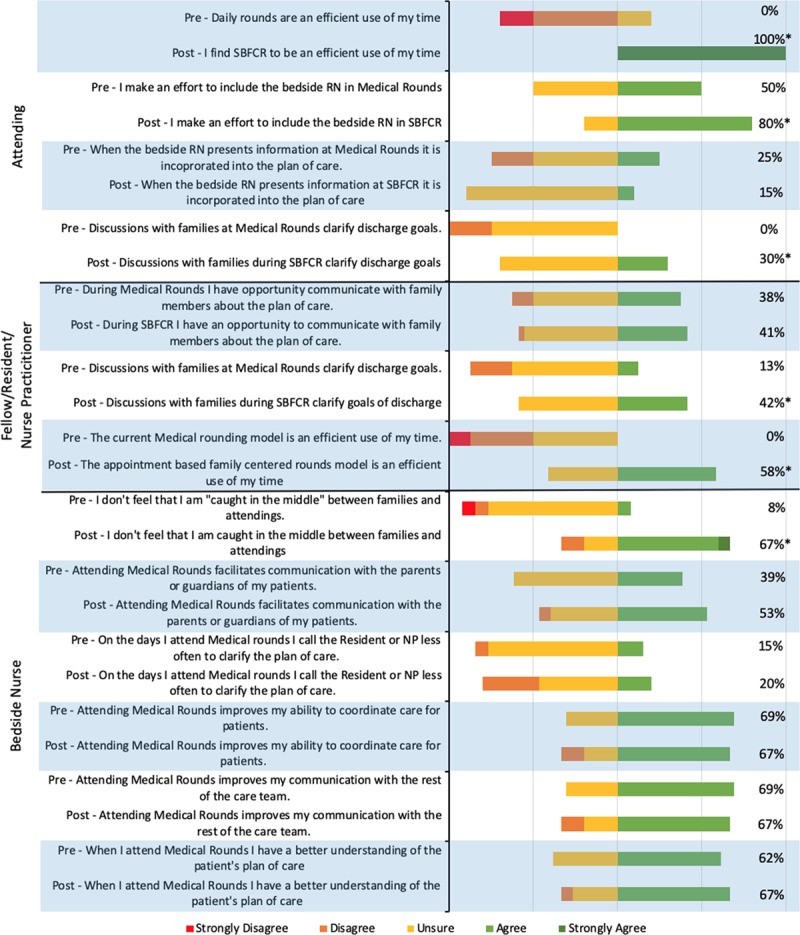
Provider and nurse satisfaction—Pre-SBFCR compared to Post-SBFCR. N% is the percent of positive (agree or strongly agree) of all responses. *Difference is significant at *P* < 0.05. SBFCRs, schedule-based family-centered rounds.

The 9-question preimplementation survey was completed by 24 nurses, 8 residents, nurse practitioners, and fellows, and 4 faculty members with an overall response rate of 59%. A 15-question postimplementation survey, including the original 9 questions from the preimplementation survey, was completed by 15 nurses, 29 residents, nurse practitioners, and fellows, and all 10 faculty with an overall response rate of 75%. Notable themes in survey responses across the disciplines following SBFCR implementation included greater efficiency of rounds and better communication among team members and families regarding discharge preparation (Fig. 4). Most fellows, residents, and nurse practitioners felt the team had to wait less for the nurse, the interpreter, or other ancillary members to start rounds after SBFCR implementation. Most RNs agreed that attending rounds facilitated communication with families, understanding of the care plan, and care coordination. All providers and most RNs felt there was sufficient time to round on each patient. All faculty strongly agreed that SBFCR is an efficient use of their time. In the comments section, 6 residents indicated the desire to implement SBFCR on all acute care pediatric teams.

### Impact on Teaching

Seven of 10 faculty physicians observed teaching increased with SBFCR, whereas 3 felt there was a neutral effect. Fifteen of 29 trainees/nurse practitioners felt teaching had increased, whereas 7 perceived no change, and 7 disagreed with the statement that teaching had increased with SBFCR. The 7 who indicated that they disagree with “an increase in the amount of teaching during rounds” could have perceived a neutral effect or a decrease in teaching compared with the pre-SBFCR era. Brief lectures were documented by the case manager to occur in 65% of workweek rounding days during the 16 minutes reserved for transfers or teaching. At least one instance of bedside teaching, apart from the discussion of any discrepancy in physical examination findings between frontline provider and faculty, was documented in 50% of encounters.

### Family Attendance and Satisfaction

Family member(s) were present for 78% of patients over 14 months. Family presence at rounds did not change significantly between preintervention and phase I (59% versus 66%; *P* = 0.183). In phase II, with daily notification to families of the scheduled rounding time, presence at rounds increased significantly (66%–85%; *P* < 0.001). We administered a 12-item survey to 56 family members before and 37 families after phase II. All respondents agreed that “knowing when the medical team will round is helpful,” and more families knew when the medical team would round after phase II (84% versus 59%, *P* = 0.007). Families also tended to have a better understanding of the discussion during rounds (83%–93%) and feel better informed about the projected discharge date (77%–87%).

## DISCUSSION

This study demonstrates that SBFCR significantly increased RN attendance and RN participation while maintaining perceived teaching. As standard rounding scripts were implemented before phase I, we attribute this improvement to the scheduling process. Given that bedside nurses spend the most time with patients, we believe RN participation at rounds is vital for communication and optimal decision making.

Previous literature describes modest improvements in nursing attendance using hands-free devices^8,13,14^ or phone calls to notify the RN of rounds.^15^ A common barrier with these types of unscheduled notifications is that the RN may be caring for another patient or on a scheduled break and be unable to attend rounds. Aragona et al.^15^ postulated that improved coordination of patient rounding time and other nurse responsibilities would likely improve nurse attendance, and scheduling rounds was an organizational strategy identified to improve family engagement in FCR. SBFCR offers a method to achieve this coordination. In our study, keeping the order of patients was associated with increased RN attendance and participation, but starting earlier or later than the set time was not associated with fluctuations in these metrics. The nurses worked closely with the case manager to be aware of when the team was running ahead or behind the set schedule. This awareness helps explain the maintenance of high attendance despite the 47% of encounters starting >5 minutes earlier or later than the scheduled time.

Our project demonstrated that setting a schedule and using a standardized presentation format improved efficiency by minimizing information repetition while consistently addressing key care issues. Although the average rounding time/patient increased by 1 minute, it was value-added time because the checklist items were reliably discussed. Additionally, providers perceived less time spent between patients waiting for the nurse or interpreter, so rounds felt more efficient. The time invested in rounds may decrease clarifications from RNs or families afterward, which likely contributed to perceived efficiency.^5^ Similar standardization approaches to rounds have shown to significantly increase both healthcare provider’s satisfaction and quality of discussion during rounds.^16^ We also found SBFCR improved overall satisfaction with rounds for providers. High engagement and satisfaction with the rounding process, which is a major component of the inpatient workday, can have a positive effect on patient outcomes while decreasing provider burnout.^17^

Education is an essential component of the mission of teaching hospitals. Before starting SBFCR on the cardiology service, a major concern was that teaching would be negatively impacted due to the schedule’s time constraints. Despite these concerns, our survey results revealed most faculty and trainees perceived an increase in teaching. This favorable impression on teaching is consistent with prior FCR literature.^18,19^

Several limitations to our study deserve discussion. As a single institution study on one unit’s subspecialty service, findings may not be generalizable to a more heterogeneous patient population. However, we recently implemented the system on the general pediatric hospitalist service, whose patients are on several different units with excellent early results. Second, as this was a project done in phases, concurrent improvement initiatives, the Hawthorne effect, or unrecognized trends could impart biases. Third, the audit data collected by the case manager during rounds were not independently verified and did not capture between rounding wait time or any weekend data. Last, although we conducted surveys of providers and nurses before and after SBFCR, we did not use a validated tool to assess nurse-physician collaboration.^20^ Not all trainees had experienced cardiology rounds before and after SBFCR, limiting the comparison concerning the perception of teaching. The RN survey did not directly solicit feedback on overall satisfaction with the SBFCR process and focused on the perceived benefits of attending rounds.

In conclusion, SBFCR offers an innovative organizational framework to allow a team to accomplish the central tenets of family-centered rounding: medical decision making, clarifying care plans for the family and interdisciplinary team, care coordination, discharge preparation, and medical trainee teaching. Implementation of SBFCR increased RN attendance and participation during rounds at our institution, facilitated family presence at rounds, and improved provider satisfaction with rounds while preserving bedside education. Future studies should focus on the impact of SBFCR on patient safety and patient–family satisfaction.

## DISCLOSURE

The authors have no financial interest to declare in relation to the content of this article.

## ACKNOWLEDGMENTS

The improvement team would like to thank Glyn Williams, who helped design the intervention, Patria Eustaquio and Piper Church for supporting this project and implementing the daily management system for nursing attendance, and Claudia Antillon who helped with data entry for analysis. Paul Sharek contributed important insights into the development and sustainability of this project and assisted in the preparation of the manuscript.

## Supplementary Material


