# Supplementary material for: Schedule-based Family-centered Rounds: A Novel Approach to Achieve High Nursing Attendance and Participation
Source: Pediatr Qual Saf. 2020 Mar 13;5(2):e265. doi: 10.1097/pq9.0000000000000265 (PMC7190241; doi:10.1097/pq9.0000000000000265)
Supplement: Supplementary file 1 [file pqs-5-e265-s001.pdf]

Stanford MEDICINE

Stanford Children's Health | Lucile Packard Children's Hospital Stanford

Family Centered Rounds for Each Patient

Team gathers outside patient room • Begin when medical team/RN/interpreter are present

**Resident/NP**	Invite family to participate.
**Fellow**	(First day rounding on patient on 3W) Intro team & FCR process to the family.
**Attending**	What are most important issues/data to review for this patient today?
**Bedside RN**	Present introductory line; note "post op day #" if applicable.<br>Overnight events/issues in the last few hours.<br>Any specific concerns for their shift.<br>Current VS + Trends.<br>Today's weight & weight trend.<br>Total ins + outs and balance and (if applicable) last 24hrs CT output.
**Resident/NP**	Telemetry review. If no alarms, state: "No concerns on telemetry" .<br>Cardiac Exam/Other **key** exam findings (do not need ALL systems).
	**—PAUSE—**
**Case Manager**	Call Nurse and Ancillaries for <u>*next*</u> patient.
**Attending & Fellow**	Examine patient; exam teaching opportunity.
**Other R2/NP**	Show CXR on the computer in the room.
**Resident/NP**	**Assessment** and Plan of Care – by problem.<br>Name meds in active problem.<br>Report labs and interpret them in applicable problem.<br>(As needed) Central line – target d/c date.<br>Pain issues: Ask family, ***"How well is your child's pain being controlled?"*** and as needed, ***"What can we be doing better?"***<br>Target d/c date and time (am, pm) + review discharge criteria/needs.
**Fellow**	Summarize plan of care for the day.
**Bedside RN**	As needed, clarify care plan; update team on d/c teaching status.
**Case Manager**	**Go through EPIC Rounds Checklist: PAIN, CENTRAL LINE, MONITORS, TDD.**
**Resident/NP**	Ask if family understands and agrees with plan & DC goals. Any questions?<br>Offer to return if discussion > 2 min required.<br>Thank family for participating.
