# Supplementary material for: Schedule-based Family-centered Rounds: A Novel Approach to Achieve High Nursing Attendance and Participation
Source: Pediatr Qual Saf. 2020 Mar 13;5(2):e265. doi: 10.1097/pq9.0000000000000265 (PMC7190241; doi:10.1097/pq9.0000000000000265)
Supplement: Supplementary file 2 [file pqs-5-e265-s002.docx]

| **What is my role?**  - Ask questions - Write down notes - Provide additional information - Share your insights about your child. - You are free to just listen  **What about after FCR?**  - It is okay if you choose not to participate in FCR…your team will still provide you with updates. - **The team will set up a time to return when discussion will exceed the appointment length of 8-10 minutes.**  **How can I prepare for home?**  - Learn and practice everyday activities such as bathing a child with a wound, lifting your child, and giving medications. - Nurses are available at all times to answer questions and can provide support when you need a break. |  |  | \| Family Centered Rounds FCR is a time when the healthcare team meets with you to coordinate your child’s care. Privacy and Confidentiality Lucile Packard Children’s Hospital Stanford is committed to keeping patient medical information private. Doctors and other staff follow Federal guidelines (HIPAA) related to patient and family privacy and will not share patient information with anyone outside of a child’s healthcare team. If you have questions, please talk with your nurse. \| \| --- \| \| \|  \|  \| \| --- \| --- \| \| |  |  | \| **You are invited to Scheduled Family Centered Rounds:**  **PCU 200 Cardiology** \| \| --- \|   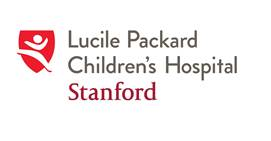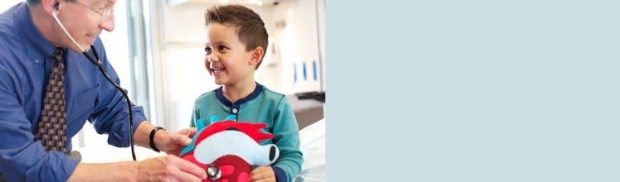 |
| --- | --- | --- | --- | --- | --- | --- | --- | --- | --- | --- | --- |

| What is Family Centered Rounds (FCR)?  A time when your child’s healthcare team meets with you for **8-10 minutes** to:   - Hear your perspective - Review your child’s current health status - Share new information - Develop a daily plan of care - Discuss goals for discharge from the hospital - Coordinate your child’s care - Hear team discuss teaching points about conditions that may be related to your child’s illness   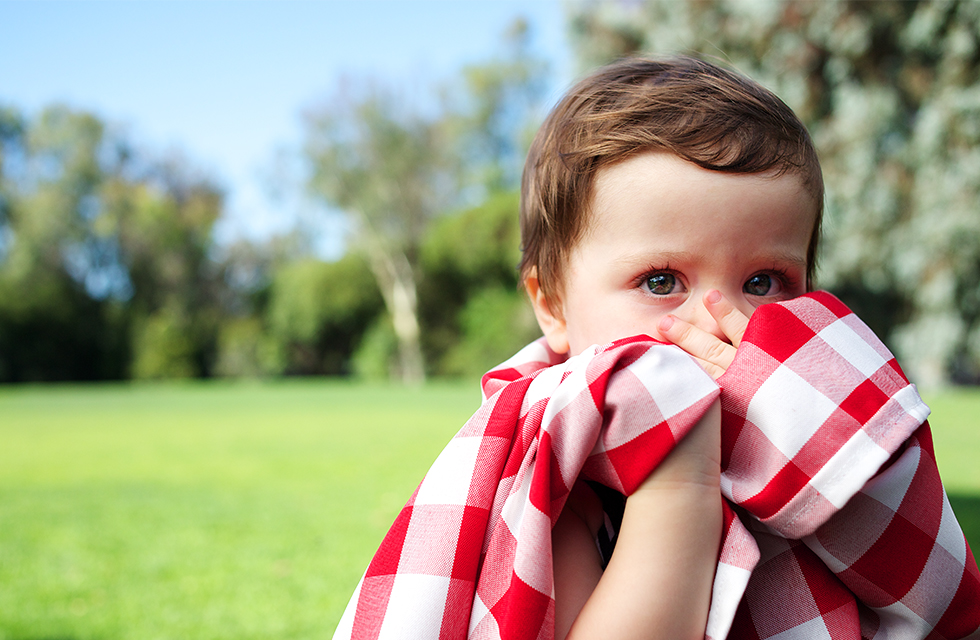 |  | **“It is awesome. It is all-inclusive. You can’t believe how great it is.”**  **“I like that they value my opinions and listen to me. FCR is very efficient and they include me. I get all the information I need every day. I am so much happier with my experience here…”** | **Why is FCR Important?**Improves communication among team members  - Allows the patient and family to partner with the team  **Who Participates?**  - Patient and Family - Doctors (Attending, Fellow, Resident) - Nurse Practitioners - Nurses (Charge, Bedside) - Case Manager - Dietician - Pharmacist - Ancillary Team Members – as needed   - Interpreter, Social Worker   - Therapists (Occupational, Physical, Respiratory) |  |  | 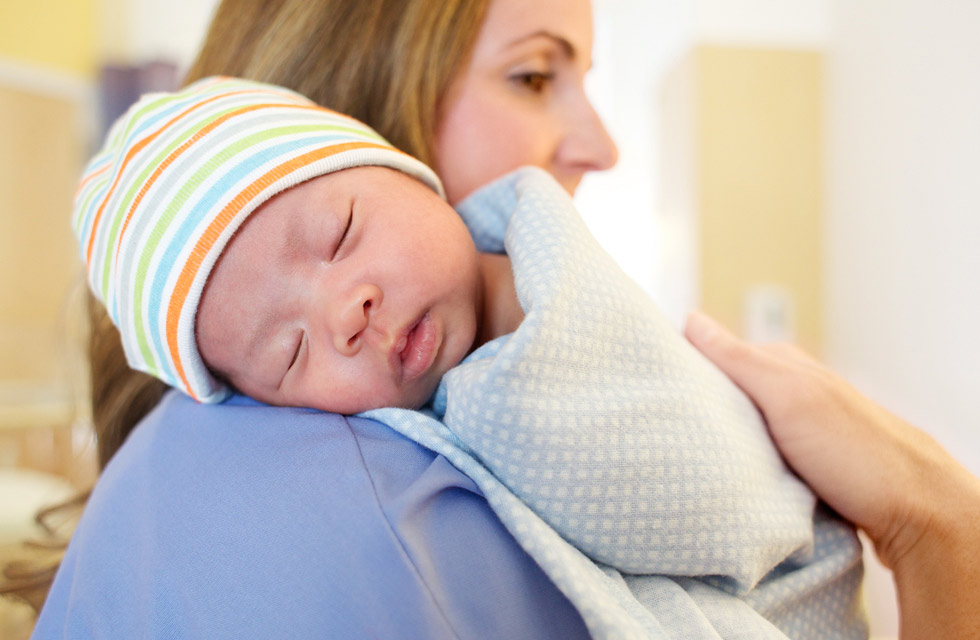**When and Where?**Monday-Thursday 8:35 am – 11:00 am  - Friday – Sunday: **9:00-11am** - You can choose to meet in your child’s room or in the hallway - Your nurse will inform you of the rounds start time and this will be noted on paper at bedside table each morning. - For parents staying outside the hospital, you can call and ask when your child’s rounding window will be after 7:30am on the day of rounds.  **More Questions?**Ask your Bedside Nurse, Doctor, Nurse Practitioner or Social Worker |
| --- | --- | --- | --- | --- | --- | --- |

# **More Questions?**

***Our cardiology team members can help answer your questions.***
Directing your questions to the right person can help you get the best answer quickly. Your child’s bedside nurse is always able to help get the right answer to your question if the team members below are not immediately available.

| **Question** | **Team Member** |
| --- | --- |
| - Will my child need any additional surgeries? - What is my child’s long-term prognosis? | Pediatric cardiologist |
| - How long will my child need to take the prescribed medications? - What can I expect during this hospital stay? - What signs or symptoms should I watch for? - Are there any physical activity restrictions? | Nurse practitioner |
| - What medications is my child currently taking? When are they given? - How do I care for the wound and bathe my child? - How do I administer chest physiotherapy? | Bedside nurse |
| - How do I learn how to use the medical supplies that are being sent home with me? - When will the supplies be delivered? | Case manager |
| - How do I use a nebulizer machine? - How do I give respiratory treatments? | Respiratory therapist |
| - How can I arrange for a place to stay while my child is hospitalized? - What resources are available if I need financial or emotional support? | Social worker |
| - How can I help my child cope with this hospitalization? - How can I help my other children cope? | Child life specialist |
| - What formula is best for my child? - How much should my child eat? | Nutritionist |
