# Supplementary material for: Schedule-based Family-centered Rounds: A Novel Approach to Achieve High Nursing Attendance and Participation
Source: Pediatr Qual Saf. 2020 Mar 13;5(2):e265. doi: 10.1097/pq9.0000000000000265 (PMC7190241; doi:10.1097/pq9.0000000000000265)
Supplement: Supplementary file 3 [file pqs-5-e265-s003.pdf]

Patient	Unit	Room	Nurse	Start Time	Service	Language	Discharge	DC Before Rounds?	High Priority?	Adjust Duration	Duration	Comments/Consults
PATIENT A	PCU 374	3752-A	RN A	08:35	PACT	English	Today	No	No	− 0 +	10	
PATIENT B	PCU 374	3740-A	RN D	08:45	PACT	English	> 48 hrs	No	No	− 0 +	10	DC Today
PATIENT C	PCU 374	3746-B	RN D	08:55	PACT	Spanish	Today	No	No	− 0 +	10	
PATIENT D	PCU 374	3748-A	RN B	09:05	PACT	Spanish	> 48 hrs	No	No	− 0 +	10	
PATIENT E	PCU 374	3760-A	RN A	09:15	PACT	English	> 48 hrs	No	No	− 0 +	10	Poss DC today late
PATIENT F	PCU 374	3746-A	RN C	09:25	PACT	English	> 48 hrs	No	No	− 0 +	10	
PATIENT G	PCU 374	3744-A	RN E	09:35	PACT	English	> 48 hrs	No	No	− 0 +	10	
PATIENT H	PCU 374	3742-A	RN C	09:45	PACT	Spanish	> 48 hrs	No	No	− 0 +	10	
PATIENT I	PCU 374	3754-A	RN E	09:55	PACT	English	> 48 hrs	No	No	− 0 +	10	
PATIENT J	PCU 374	3756-A	RN F	10:05	PACT	Spanish	> 48 hrs	No	No	− 0 +	10	
PATIENT K	PCU 374	3762-A	RN D	10:15	PACT	English	> 48 hrs	No	No	− 0 +	10	
PATIENT L	PCU 374	3764-A	RN A	10:25	PACT	English	> 48 hrs	No	No	− 0 +	10	
PATIENT M	PCU 374	3766-B	RN B	10:35	PACT	English	> 48 hrs	No	No	− 0 +	10	
CVICU Transfer/Activity Slot				10:45						− 0 +	16	
PATIENT N	PCU 374	3768-A	RN C	11:01	Non-PACT	English	> 48 hrs	No	No	− 0 +	8	Poss Dc today late
PATIENT O	PCU 374	3768-B	RN F	11:09	Non-PACT	English	> 48 hrs	No	No	− 0 +	8	
PATIENT P	PCU 374	3770-A	RN B	11:17	Non-PACT	English	> 48 hrs	No	No	− 0 +	8	
PATIENT Q	PCU 374	3770-B	RN F	11:25	Non-PACT	English	> 48 hrs	No	No	− 0 +	8	
PATIENT R	PCU 374	3766-A	RN E	11:33	Non-PACT	English	> 48 hrs	No	No	− 0 +	8	
PATIENT S	PCU 374	3756-B	RN F	11:41	Non-PACT	English	> 48 hrs	No	No	− 0 +	8	
